# Adjuvant chemoradiotherapy instead of revision radical resection after local excision for high-risk early rectal cancer

**DOI:** 10.1186/s13014-016-0692-9

**Published:** 2016-09-05

**Authors:** Jae-Uk Jeong, Taek-Keun Nam, Hyeong-Rok Kim, Hyun-Jeong Shim, Yong-Hyub Kim, Mee Sun Yoon, Ju-Young Song, Sung-Ja Ahn, Woong-Ki Chung

**Affiliations:** 1 Department of Radiation Oncology, Chonnam National University Medical School, Hwasun-eup, Hwasun-gun, Jeonnam South Korea; 2 Department of Surgery, Chonnam National University Medical School, Hwasun-eup, Hwasun-gun, Jeonnam South Korea; 3 Department of Hemato-Oncology, Chonnam National University Medical School, Hwasun-eup, Hwasun-gun, Jeonnam South Korea

**Keywords:** Local excision, Early rectal cancer, Adjuvant chemoradiotherapy

## Abstract

**Background:**

After local excision of early rectal cancer, revision radical resection is recommended for patients with high-risk pathologic stage T1 (pT1) or pT2 cancer, but the revision procedure has high morbidity rates. We evaluated the efficacy of adjuvant concurrent chemoradiotherapy (CCRT) for reducing recurrence after local excision in these patients.

**Methods:**

Eighty-three patients with high-risk pT1 or pT2 rectal cancer underwent postoperative adjuvant CCRT after local excision. We defined high-risk features as pT1 having tumor size ≤3 cm, and/or resection margin (RM) ≤3 mm, and/or lymphovascular invasion (LVI), and/or non-full thickness excision such as endoscopic mucosal resection (EMR) or endoscopic submucosal dissection (ESD), or unknown records regarding those features, or pT2 cancer. Radiotherapy was administered with a median dose of 50.4 Gy in 1.8 Gy fraction size over 5–7 weeks. Concurrent 5-fluorouracil and leucovorin were administered for 4 days in the first and fifth weeks of radiotherapy.

**Results:**

The median interval between local excision and radiotherapy was 34 (range, 11–104) days. Fifteen patients (18.1 %) had stage pT2 tumors, 22 (26.5 %) had RM of ≥3 mm, and 21 (25.3 %) had tumors of ≥3 cm in size. Thirteen patients (15.7 %) had LVI. Transanal excision was performed in 58 patients (69.9 %) and 25 patients (30.1 %) underwent EMR or ESD. The median follow-up was 61 months. The 5-year overall survival (OS), locoregional relapse-free survival (LRFS), and disease-free survival (DFS) rates for all patients were 94.9, 91.0, and 89.8 %, respectively. Multivariate analysis did not identify any significant factors for OS or LRFS, but the only significant factor affecting DFS was the pT stage (*p* = 0.027).

**Conclusions:**

In patients with high-risk pT1 rectal cancer, adjuvant CCRT after local excision could be an effective alternative treatment instead of revision radical resection. However, patients with pT2 stage showed inferior DFS compared to pT1.

## Background

Radical surgery has been the standard of treatment for patients with rectal cancer and adjuvant concurrent chemoradiotherapy (CCRT) is often recommended in order to decrease the risk of recurrence for patients with locally advanced rectal cancer. A randomized controlled study comparing adjuvant CCRT with neoadjuvant CCRT has showed more sphincter preservation, a decreased rate of pelvic recurrence, and a lower incidence of treatment-related toxicities in the neoadjuvant CCRT group [1]. In selected cases, local excision after neoadjuvant CCRT had comparable oncologic outcomes to radical surgery, with fewer complications [2], and local recurrence rates of <20 % have been reported in patients with stage T2 tumors after local excision with neoadjuvant CCRT [3, 4].

Transanal excision (TAE) can be performed as an initial treatment in patients with early rectal cancers who have well to moderately differentiated stage T1 tumors that are <30 % of the circumference, <3 cm in size, mobile, non-fixed, and without lymphovascular invasion (LVI) or perineural invasion [5, 6]. Local excision should be performed in patients with no evidence of lymphadenopathy on pretreatment imaging because lymph node metastasis has been reported at rate of 17 to 31 % in patients with pathologic stage T1 (pT1) and pT2 rectal cancers [7].

TAE alone has been associated with a higher instance of local recurrence (2.7 vs. 13.2 %, *p* = 0.001) and inferior disease-specific survival when compared to radical surgery for tumors with high-risk pathologic features [5], and revision radical resection is often necessary after local excision for patients with these tumors [8, 9]. Radical surgery has a 2–3 % perioperative mortality rate and 20–30 % complication rate, including bowel, bladder, and sexual dysfunction and permanent colostomy [10]. Local excision and adjuvant CCRT have been attempted instead of revision radical surgery in order to avoid major morbidities, and local excision with adjuvant CCRT may offer better oncologic outcomes than local excision alone [11–14]. However, the efficacy of adjuvant CCRT after local excision remains controversial, and evidence is lacking, as there are few published reports so far.

We present a retrospective single-center analysis of survival outcomes in a relatively large cohort to investigate the role of adjuvant CCRT after local excision as an alternative to revision radical surgery in patients with early stage high-risk rectal cancers.

## Methods

### Patient eligibility

Patients who received adjuvant CCRT after local excision of rectal cancer between January 2004 and December 2012 were eligible for inclusion. Clinical imaging before local excision included abdominal-pelvic computed tomography (CT) and/or pelvic magnetic resonance imaging (MRI), and chest CT. Tumor stage was classified in accordance with the American Joint Committee on Cancer staging system, seventh edition. Eligibility criteria included a histological diagnosis of adenocarcinoma, pT1, after local excision of the primary rectal cancer, with high-risk features including tumor size ≥3 cm, and/or resection margin (RM) ≤3 mm, and/or LVI, and/or non-fullthickness excision such as endoscopic mucosal resection (EMR) or endoscopic submucosal dissection (ESD), or unknown records regarding those features, or pT2 cancer. Patients with no evidence of distant metastasis, no previous history of other cancers, and no previous pelvic radiotherapy were eligible in this analysis. Institutional review board at our institute approved this study.

### Adjuvant treatments

Radiotherapy was performed with 6-MV or 10-MV X-rays via three- or four-field box technique for two-dimensional (2D) or three-dimensional (3D) conformal radiotherapy. The superior border of the 2D field was the sacral promontory, the inferior border was 3 cm distal to the tumor or the inferior obturator foramen, the lateral border was 1 cm from the bony pelvis, and the anterior and posterior borders of the lateral portals were at the posterior symphysis pubis and at 1 cm behind the anterior bony sacrum. The clinical target volume (CTV) for 3D conformal radiotherapy included the primary tumor bed, the mesorectum, and the presacral, obturator, internal iliac, and distal common iliac lymph nodes. The PTV was defined as 0.5 cm margin around the CTV in all directions. The planned dose to PTV was up to 45.0 Gy in 1.8 Gy fraction size over 5 weeks and an additional boost dose of 5.4 Gy was administered to tumor bed. A concurrent chemotherapy regimen of 5-fluorouracil (5-FU; 425 mg/m^2^/day) and leucovorin (LV; 20 mg/m^2^/day) was administered for 4 days during weeks 1 and 5 of radiotherapy, and after completion of the CCRT, adjuvant chemotherapy consisting of 5-FU (425 mg/m^2^/day) and LV (20 mg/m^2^/day) administered for 5 days every 4 weeks, for up to four cycles, was recommended.

### Follow-up and statistical analysis

Regular follow-up visits were scheduled at 3-month intervals following completion of radiotherapy, including sigmoidoscopy and abdominal-pelvic CT or pelvic MRI for up to 2 years, and at 4- to 6-month intervals for the next 3 years. Chest CT was also scheduled to be checked at 6-month intervals. Treatment failure was defined as showing newly developed soft tissue mass or lymph node on CT or MRI, and pathologic confirmation was required for diagnosis of local recurrence. Overall survival (OS) was defined as the time from the start of CCRT to death from any cause or the last follow-up. Locoregional relapse-free survival (LRFS) was defined as survival without locoregional recurrence from the start date of CCRT to the date of last follow-up or death from any cause. Disease-free survival (DFS) was defined as the time from the start of therapy to the date of treatment failure or last follow-up. Survival rates were calculated up to 60 months of follow-up in order to remove bias due to death from natural causes among elderly patients surviving more than 60 months.

The Kaplan-Meier method was used to plot survival curves, which were analyzed using log-rank tests to determine prognostic factors in univariate analysis. A Cox regression model was used to identify prognostic factors in the multivariate analysis. All statistical analyses were performed using SPSS, version 19.0 (SPSS Inc., Chicago, IL, USA). A statistically significant difference between groups was indicated at *p* < 0.05. Adverse events were defined according to the National Cancer Institute Common Terminology Criteria for Adverse Events (CTCAE), version 4.0.

## Results

### Patient characteristics

**Fig. 1:**
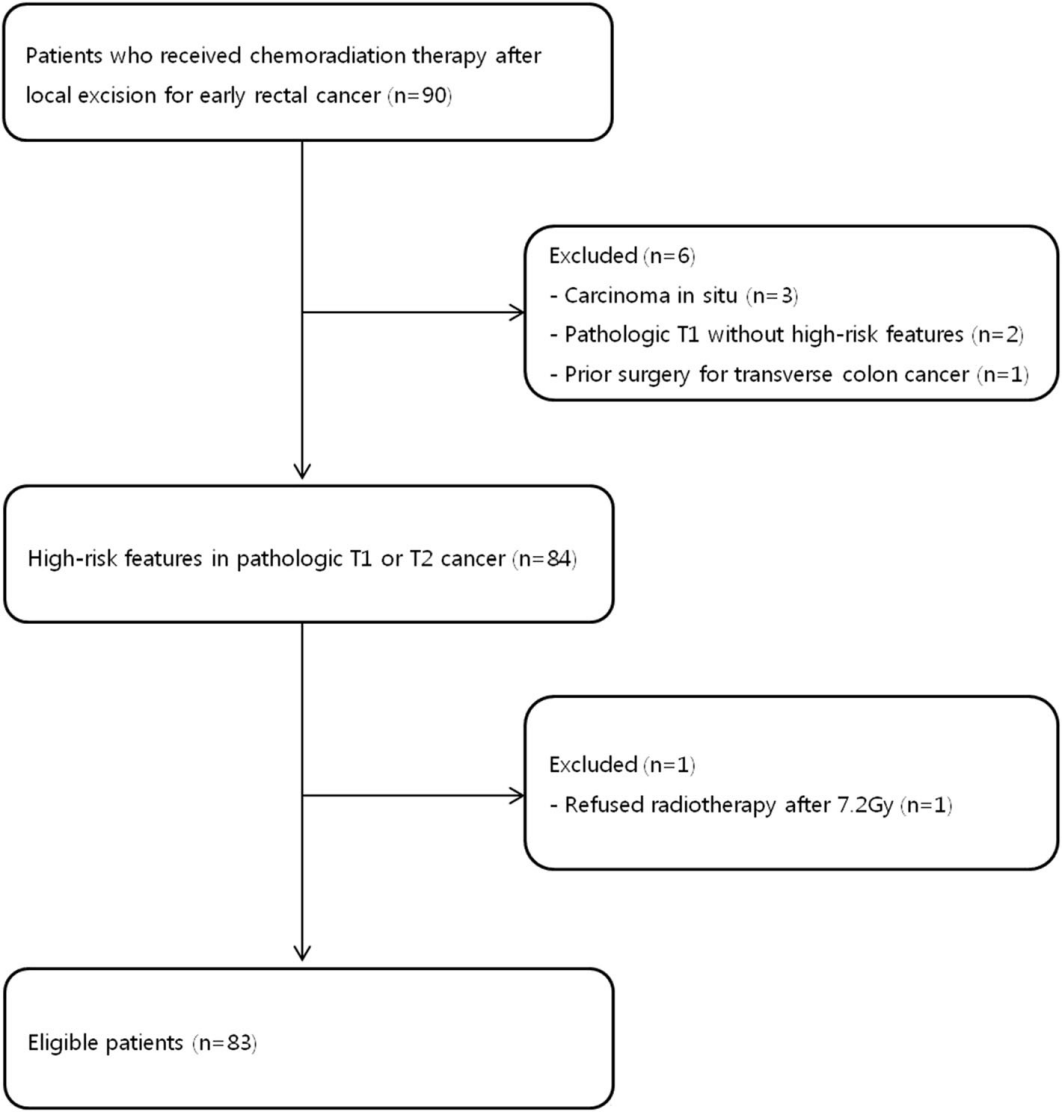
Eligible patients who received adjuvant chemoradiotherapy after local excision for early rectal cancer with high-risk features

During the study period, a total of 90 patients received adjuvant CCRT after local excision for early rectal cancer. Of these, seven patients were excluded, including three who had carcinoma in situ, two who did not have any high-risk features, one who chose to discontinue RT after 7.2 Gy in four fractions, and one who had colectomy for transverse colon cancer 1 month before TAE for the rectal tumor (Fig. 1). Thus, 83 patients (57 men and 26 women, median age 66 years) were included in the study (Table 1). The median interval between local excision and the start of CCRT was 34 days (range 11–104). Fifteen patients (18.1 %) had stage pT2 tumors, 22 (26.5 %) had RM of ≤3 mm, and 21 (25.3 %) had tumors of ≥3 cm in size. Thirteen patients (15.7 %) had LVI. TAE was performed in 58 patients (69.9 %) and 25 patients (30.1 %) underwent EMR or ESD. The median radiotherapy dose was 50.4 Gy (range 39.6–59.4 Gy). There were two patients in the study group (2.4 %) who chose not to receive chemotherapy, and therefore had RT only; there were 14 patients (16.9 %) who consented to an additional course of adjuvant chemotherapy following the CCRT: 1 of these received five cycles of oral therapy with tegafur and uracil (300/672 mg/m^2^/day) for 4 weeks per cycle due to travel difficulties; all of the others were to receive the regimen described above (Table 1).

**Table 1:** Patient, tumor, and treatment characteristics

Characteristics	Number of patients	Percent
Sex		
Male	57	68.7
Female	26	31.3
Age (years)		
Median	66	
Range	32–84	
CEA (ng/mL)		
Median	2.36	
Range	0.5–18.32	
Unknown	9 patients	
Tumor size (cm)		
Median	2	
Range	0.6–5.2	
Tumor location (cm from anal verge)		
Median	5	
Range	2–20	
T-stage		
1	68	81.9
2	15	18.1
Differentiation		
Well differentiated	46	55.4
Moderately differentiated	32	38.6
Poorly differentiated	2	2.4
Unknown	3	3.6
Lymphovascular invasion		
No	41	49.4
Yes	13	15.7
Unknown	29	34.9
Perineural invasion		
No	42	50.6
Yes	1	1.2
Unknown	40	48.2
Resection margin		
> 3 mm	3	3.6
≤ 3 mm	22	26.5
Unknown	58	69.9
Local excision		
Endoscopic mucosal resection	21	25.3
Endoscopic submucosal dissection	4	4.8
Transanal excision	58	69.9
Radiotherapy (Gy)		
Median	50.4	
Range	39.6–59.4	
Concurrent chemotherapy		
No chemotherapy	2	2.4
FL	79	95.2
FP	2	2.4
Adjuvant chemotherapy		
No chemotherapy	69	83.1
5-FU	13	15.7
oral 5-FU prodrug	1	1.2

*FL* 5-fluorouracil + leucovorin, *FP* 5-fluorouracil + cisplatin, *5-FU* 5-fluorouracil

### Survival and prognostic factors

**Fig. 2:**
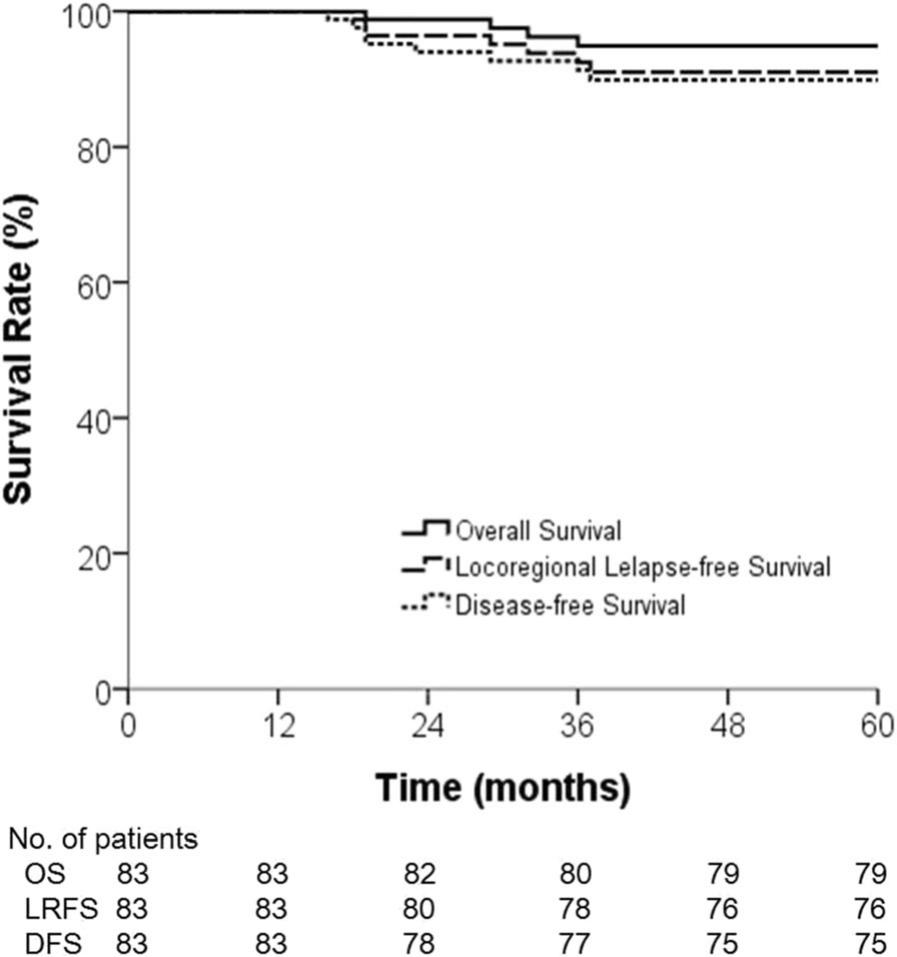
Overall survival (OS), locoregional relapse-free survival (LRFS), and disease-free survival (DFS) in entire patient cohort

At the time of the final analysis, 75 patients (90.4 %) had survived without disease and two (2.4 %) were alive with disease. The median duration of follow-up was 61 months. Five-year OS for all 83 patients was 94.9 %, with LRFS of 91 %, and DFS of 89.8 % (Fig. 2). The variables of sex, age, tumor location, serum carcinoembryonic antigen levels, tumor size, pathologic T stage, RM, LVI, type of local excision, radiation dose, and adjuvant chemotherapy were used for both univariate and multivariate analysis. Age was the only significant factor associated with OS on univariate analysis (*p* = 0.031), and this may have been because death from any cause was considered as an event when calculating survival. According to DFS, pathologic T stage showed statistical significance in univariate analysis (*p* = 0.015). On multivariate analysis, there were no significant factors for OS or LRFS (Table 2), but pathologic T stage was the only significant factor influencing DFS (*p* = 0.027).

**Table 2:** Prognostic factors related with overall survival (OS), locoregional relapse-free survival (LRFS), and disease-free survival (DFS)

	OS		LRFS		DFS	
	Univariate	Multivariate	Univariate	Multivariate	Univariate	Multivariate
(No. of patients)	5-year rate (%)	*p-*value	5-year rate (%)	*p-*value	5-year rate (%)	*p-*value
Sex		n-s		n-s		n-s
Male (57)	94.4		92.6		90.9	
Female (26)	95.8		87.6		87.6	
Age		n-s		n-s		n-s
< 70 (58)	98.2^a^		94.8		93.1	
≥ 70 (25)	86.4		81.0		81.0	
Location from anal verge		n-s		n-s		n-s
< 5 cm (33)	96.7		93.6		90.7	
≥ 5 cm (50)	93.7		89.4		89.4	
CEA level		n-s		n-s		n-s
< 3 ng/ml (49)	100		95.6		93.6	
≥ 3 ng/ml (34)^b^	87.0		84.0		84.3	
Tumor size		n-s		n-s		n-s
< 3 cm (62)	94.9		89.7		88.1	
≥ 3 cm (21)	94.7		94.7		94.7	
T-stage		n-s		n-s		0.027 (HR: 4.8, 95 % CI: 1.2–19.1)
1 (68)	95.2		93.7		93.8^a^	
2 (15)	93.3		80.0		73.3	
Resection margin		n-s		n-s		n-s
> 3 mm (3)	100		100		100	
≤ 3 mm (22)	95.5		86.4		81.8	
Unknown (58)	94.3		92.1		92.1	
LVI		n-s		n-s		n-s
No (41)	92.1		87.1		84.9	
Yes (13)	92.3		83.9		83.9	
Unknown (29)	100		100		100	
Local excision		n-s		n-s		n-s
EMR or ESD (26)	92.0		88.1		88.1	
TAE (57)	96.2		92.2		90.5	
Radiation dose		n-s		n-s		n-s
> 50.4 Gy (8)	100		100		100	
≤ 50.4 Gy (75)	94.3		90.0		88.7	
Adjuvant chemotherapy		n-s		n-s		n-s
Yes (14)	100		92.9		92.9	
No (69)	93.9		90.7		89.3	

*N-S* not significant, *LVI* lymphovascular invasion, *EMR* endoscopic mucosal resection, *ESD* endoscopic submucosal dissection, *TAE* transanal excision, *HR* hazard ratio, *CI* confidence interval, ^a^statistically significant in univariate analysis, ^b^ Nine patients of unknown CEA level were included in this subgroup

### Treatment failures and toxicities

**Fig. 3:**
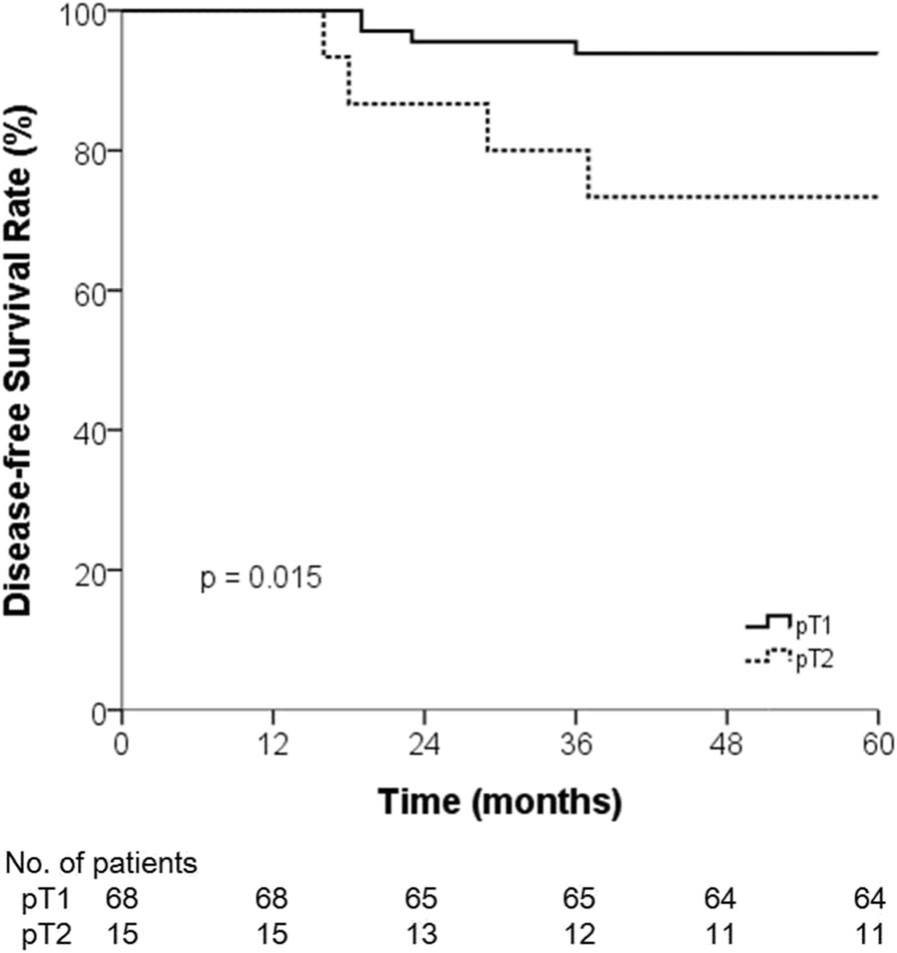
Disease-free survival according to pathologic T-stage

Two of five patients (2.4 %) with observed treatment failure had isolated local recurrences, two (2.4 %) had isolated distant recurrences, and one (1.2 %) had simultaneous local and distant recurrence. No regional treatment failures were observed in any patient. Among the five patients with local and distant recurrences, four (80 %) had stage pT2 tumors or surgical RM of ≤3 mm. Five-year LRFS for patients with stage pT1 vs. pT2 tumors was 93.7 vs. 80.0 %, respectively, but this difference was not significant (*p* = 0.091). However, the 5-year DFS did differ significantly, at 93.8 % for patients with stage T1 tumors and 73.3 % for those with stage T2 tumors (*p* = 0.015) (Fig. 3). Regarding the two patients who had isolated local failure, one patient received intensity-modulated re-irradiation with 5-FU after re-excision and the other underwent salvage laparoscopic abdominoperineal resection with adjuvant chemotherapy and they had survived without disease at the last follow-up. The patient with loco-distant recurrence had progressive disease despite salvage chemotherapy, and one of the two patients who had isolated distant recurrences was alive after salvage liver metastasectomy, while the other died of disease (Table 3). Grade 2 proctitis occurred in nine patients (10.8 %), and no grade 3 or higher acute toxicities developed. There were no patients who had Grade 2 or higher late toxicities. Nine patients (10.8 %) showed proctitis finding without any symptom on regular follow-up proctoscopy. There were no patients who had symptom relating anal sphincter dysfunction.

**Table 3:** Characteristics of patients with treatment failure

Sex	Age	Initial CEA	pT	Excision	Margin	Radiation dose	Disease-free interval (Months)	Failure site	Salvage treatment	Months after recurrence	Last status
Female	69	2.11	2	TAE	1.2 mm	50.4 Gy	18	Lt. perirectal area (IFF)	Laparoscopic mass excision + CCRT	30	Alive with disease
Female	76	2.24	2	TAE	unknown	50.4 Gy	37	rectum (IFF)	Laparoscopic APR+ 5-FU	57	Alive without disease
Male	69	4.75	1	EMR	1 mm	50.4 Gy	19	rectum (IFF), liver, lung	Capecitabine	32	Alive with disease
Male	68	2.04	2	TAE	0 mm	50.4 Gy	16	liver	liver S6 segmentectomy	87	Alive without disease
Male	67	unknown	1	TAE	unknown	48.6 Gy	23	liver	No	9	Dead with disease

*CEA* carcinoembryonic antigen, *pT* pathologic T-stage, *TAE* transanal excision, *IFF* in-field failure, *CCRT* concurrent chemoradiotherapy, *APR* abdominoperineal resection, *5-FU* 5-fluorouracil, *EMR* endoscopic mucosal resection

## Discussion

In this study, adjuvant CCRT after local excision was shown to have acceptable oncologic outcomes without severe complications in patients with early rectal cancers that exhibited known high-risk features for locoregional recurrence. Five-year LRFS was 91 % and DFS was 89.8 %. There were no significant factors related to OS on multivariate analysis. Neither univariate nor multivariate analysis identified significant factors associated with LRFS in the study, but the only significant factor affecting DFS was the pathologic T stage. This result implies that adjuvant CCRT could allow excellent tumor control for patients with high-risk stage T1 tumors. However, the patients with pT2 tumors survived significantly shorter DFS in this study. In the non-randomized prospective trial, 51 patients with T2 cancers received CRT after local excision, and 7/51 (14 %) experienced isolated local recurrence with 4 years median follow-up [15]. But more patients (18 %) had local recurrence in the updated results with more than 7 years median follow-up [16].

Local excision of early-stage rectal cancers is becoming a more common treatment approach [12]. However, patients may be at higher risk of recurrence after local excision compared to conventional radical resection procedures. Nash et al. reported higher recurrence rates in patients who had local excision compared with those who underwent radical resection for T1 rectal cancer (13.2 vs. 2.7 %, *p* = 0.001) [5], and local excision alone for stage pT2 tumors has been associated with a recurrence rate of up to 37 %, requiring salvage by radical resection where possible [17]. Adjuvant CCRT after local excision of stage T1 and T2 tumors has been associated with reduced local recurrence rates in several previous studies [11–14]. In one of these, the local recurrence rate among patients with stage T2 tumors who received adjuvant radiotherapy was 9 vs. 36 % among those who did not have adjuvant treatments [13]. In another, among 27 T1 patients, four of 17 patients with stage T1 tumors who had local excision without adjuvant therapy developed local recurrence (24 %) while remaining 10 patients with adjuvant CCRT did not [14].

The benefits of local excision may include reduced overall morbidity, avoidance of permanent colostomy instances, reduced mortality, and shorter hospital stay [18]. Morbidity rates of 5.6 vs. 14.5 % (*p* < 0.001) have been reported for patients undergoing local excision vs. radical resection for the initial treatment of stage T1 tumors [19]. However, salvage surgery for recurrence after initial local excision might result in poor oncologic outcomes and high morbidity [20]. In our study, only three of 83 patients (3.6 %) had local recurrence and none had severe surgical morbidities, such as are seen more frequently after radical resection. However, local excision alone cannot provide information on the regional lymph node status, and there is a known 5–10 % risk of occult lymph node metastases in patients with stage T1 tumors and a 20–35 % risk in those with stage T2 lesions [7]. The finding that there were no regional recurrences among the patients in our study suggests that adjuvant CCRT after local excision may have decreased the risk of regional recurrence by controlling regional subclinical disease.

There are several well-known risk factors for recurrence after local excision [21]. Stage T2 lesions have a higher rate of local recurrence compared with T1 lesions [19], and radical surgery or adjuvant treatment should be routine for patients with stage pT2 tumors after local excision. Patients with stage pT2 tumors who were treated with local excision followed by RT have been shown to have improved OS compared to those who have local excision only [12]. Rackley et al. recommend radical resection instead of local excision with adjuvant therapy for patients with stageT2 tumors because of inferior local control compared to T1 disease [22]. However, they do recommend local excision with adjuvant radiotherapy as a good alternative for patients with stage pT2 tumors who are not candidates for revision radical surgery. Failure to obtain a clear RM is also a well-known risk factor for local recurrence. Gopaul et al. reported that the RM was a significant factor in local treatment failure in patients treated with local excision [13]. The National Comprehensive Cancer Network® guideline recommends RM of >3 mm for TAE [23]. In our study, LRFS and DFS were worse among 22 patients with RM of ≤3 mm compared to those with RM of >3 mm, but the difference was not significant (Table 2).

Radiation doses may be varied according to the extent of the RM. In one series, a dose of 50–56 Gy was recommended in general, but if the RM was <3 mm, the dose was raised to 59.4–65 Gy. One in 33 patients with margins of <3 mm and one in 18 patients with margins of ≥3 mm had locoregional recurrence [11]. In our study, all three patients with local recurrence had radiation doses of 50.4 Gy, but they had stage pT2 tumors or RM of ≤3 mm (Table 3). There were no treatment failures among eight patients who received doses of >50.4 Gy. Among these eight patients, four (50 %) had stage pT2 tumors or RM of ≤3 mm, and all received doses of 54.0 Gy,2 having LVI received 52.2 Gy and 54.0 Gy, respectively, and no details on RM were available for the remaining two, who received 59.4 Gy and 54.0 Gy, respectively.

There are some limitations to this study. First, this study is limited by the retrospective nature and the study period spanned 9 years. During that period, there might be some inherent biases among patient or treatment characteristics. We could not obtain pathological information regarding high-risk pathologic features such as LVI or RM for many patients, as shown in Table 1. However, we classified these patients as having potentially high-risk features, considering that referral by the surgeon for adjuvant CCRT might be due to the surgeon's observation of potential high-risk features. Secondly, 14 patients (16.5 %) received adjuvant chemotherapy after completion of adjuvant CCRT. However, there were but no significant differences in clinicopathological features between patients who received adjuvant chemotherapy and those who did not (data not shown).

## Conclusion

We found adjuvant CCRT after local excision could be an effective alternative treatment instead of revision radical resection in patients with high-risk pT1 rectal cancer. However, patients with pT2 stage showed inferior DFS compared to pT1.

## Abbreviations

2D: Two-dimensional
3D: Three-dimensional
5-FU: 5-fluorouracil
CCRT: Concurrent chemoradiotherapy
CI: Confidence interval
CT: Computed tomography
DFS: Disease-free survival
EMR: Endoscopic mucosal resection
ESD: Endoscopic submucosal dissection
HR: Hazard ratio
LRFS: Locoregional relapse-free survival
LV: Leucovorin
MRI: Magnetic resonance imaging
N-S: Not significant
OS: Overall survival
PNI: Perineural invasion
pT: Pathologic tumor stage
RM: Resection margin
TAE: Transanal excision
